# A Fundamental Requisite

**Published:** 1875-05

**Authors:** 


### ARTICLE XIII.

### *A Fundamental Requisite.*

While it must be admitted that a knowledge of anatomy, physiology, pathology, and therapeutics is required in the conservation of the dental organs, and while, also, as must be equally apparent, artistic culture is every way desirable in the prosthetics of dentistry, it is nevertheless evident that the first absolute and indispensable requisite of a dental practitioner is a perception of the mechanics of the calling and manipulative dexterity.

There is danger that in the laudable ambition to justify the doctorate by medical culture the necessity of handicraft may be to a great extent neglected. It seems indeed difficult to prevent the adoption of extreme views in one direction or another on this subject. "The profession has been too much engrossed with the mere mechanics of the calling," says one. "The exaggeration of the necessity for a medical education threatens to fill our ranks with incompetents, so far as manual skill is concerned," says another. "He can remove a tumor or make a resection, but he cannot plug a tooth." "He can make a handsome filling, but that is all he knows." "He can diagnose skillfully, but he cannot correct the trouble unless it is to be met by a prescription." "He is a first-class jeweler, but has no more perception of facial requirement than any other jeweler." These and like criticisms show not only the one-sidedness of the practitioner, but generally, and as surely, an equal failure on the part of the critic to comprehend the circle of the requirements. Hypertrophy of the gums from stomatitis, referred by a medical man to a dentist, because it was not in *his* line; swelling of the face from a dead pulp and consequent alveolar abscess, referred by a dentist to the family physician, as erysipelas, because that was out of *his* line. Cases such as these show how desirable it is that a dentist should be more than a mechanic. On the other hand, fillings which can be removed with a tooth-pick, or dentures whose artificialtiy (to say nothing of their inefficiency) shocks every beholder, demonstrate that he should be something besides a doctor.

Half-knowledge in any vocation is deplorable, and yet how few in the profession of dentistry have secured the reputations of masters!

"Can you commend me to a thoroughly reliable dentist in ——?" was asked of a practitioner in the presence of the writer a short time since.

"My answer will depend upon the character of the service required," was the reply. "If you desire an artificial plate in the highest style of the art, I would name one gentleman; if you desire first-class fillings, another; but if medical advice or surgical interference in regard to abnormal conditions is sought, still another." Alas, how few there are who "fill the bill!" Yet, after all, however discreditable it may be to lack knowledge rightfully assumed as belonging to the calling, there is no lack so deplorable as that of the manual dexterity to perform the operations which are and must continue to be the staple of dental practice. If, where all are important, we must choose what particular qualification may be ignored, manipulative skill must be the last to be slighted. In the discussion of dental educational problems, it should be remembered that no medical, surgical, or æsthetical culture will compensate for the want of manual skill. Any preparation which fails to develop a high order of mechanical dexterity fails in that which is the first requisite of the dentist, as indeed it is that of the surgeon at large.

What if one can stand a creditable examination upon the pathology, diagnosis, prognosis, and treatment of all oral lesions; or can perform any surgical operation required thereby, if he cannot, when desirable, make an elegant and permanent filling? Wherever educated or graduated, such a dentist, no matter what his other qualifications, is not worthy of the name.

To natural and acquired manipulative ability, as the first requisite, should therefore be *added* the broader culture which has been indicated. The substitution of it will not meet the requirements. Every young man proposing the study of dentistry, with a view to its practice, should be instructed that, while the theory is based on principles which belong alike to every specialty of medicine, its practice necessarily involves an apprehension, to no mean extent, of mechanical laws, and includes the manual culture required to actualize the dictates of the will.—*Editorial in Dental Cosmos.*

